# Effect of Black Cumin Cake Addition on the Chemical Composition, Glycemic Index, Antioxidant Activity, and Cooking Quality of Durum Wheat Pasta

**DOI:** 10.3390/molecules27196342

**Published:** 2022-09-26

**Authors:** Ada Krawęcka, Aldona Sobota, Eva Ivanišová, Ľuboš Harangozo, Veronika Valková, Ewelina Zielińska, Agata Blicharz-Kania, Beata Zdybel, Sylwia Mildner-Szkudlarz

**Affiliations:** 1 Department of Plant Food Technology and Gastronomy, University of Life Sciences in Lublin, Skromna 8 Street, 20-704 Lublin, Poland; 2 Institute of Food Sciences, Faculty of Biotechnology and Food Science, Slovak University of Agriculture in Nitra, Tr. A. Hlinku 2, SK-949 76 Nitra, Slovak Republic; 3 AgroBioTech Research Centre, Slovak University of Agriculture in Nitra, Tr. A. Hlinku 2, SK-949 76 Nitra, Slovak Republic; 4 Department of Analysis and Evaluation of Food Quality, University of Life Sciences in Lublin, Skromna 8 Street, 20-704 Lublin, Poland; 5 Department of Biological Bases of Food and Feed Technologies, University of Life Sciences in Lublin, Głęboka 28 Street, 20-612 Lublin, Poland; 6 Institute of Food Technology of Plant Origin, Poznań University of Life Sciences, 31 Wojska Polskiego St., 60-624 Poznań, Poland

**Keywords:** enriched pasta, functional food, by-products, antioxidants

## Abstract

Pasta is a good carrier for plant enrichment substances due to its popularity among consumers. The purpose of the study was to investigate the functional potential and optimize the recipe of pasta made from durum semolina with the addition of black cumin cake at the level of 5, 10, 15, 20, and 25%. The use of black cumin cake resulted in a statistically significant (*p* ≤ 0.05) increase in the content of protein, fat, ash, and fiber, including both the insoluble and soluble fractions. A reduction in the digestible carbohydrate content, in vitro starch hydrolysis index (HI), was observed. Pasta with a reduced glycemic index (GI) compared to the semolina control was obtained. The content of polyphenols, including flavonoids, in the cake-enriched pasta increased significantly (*p* ≤ 0.05), which resulted in higher antioxidant activity against DPPH. The increase in the iron content was over 2.5 times higher in the sample with the 25% addition of black cumin cake than in the control sample. The functional addition significantly (*p* ≤ 0.05) increased the loss of dry matter and influenced the cooking time of pasta.

## 1. Introduction

Black cumin (*Nigella sativa*), i.e., a representative of the *Ranunculaceae* family, comes from Africa and South-West Asia. The crops are also located in the Mediterranean regions. Black cumin is one of the medicinal plants that has been eagerly used in traditional Indian and Arabic medicine since ancient times. In addition to its medicinal value, it is valued in those regions for cultural and religious reasons [1].

Black cumin seeds contain, on average, 34% of fat, 22% of protein, 32% of carbohydrates, 8% of fiber, especially the insoluble fraction, and 4% of ash [2]. In the analysis of the chemical composition of black cumin seeds, it is worth paying attention to the high protein quality with different biological activities and qualitative composition of fat, 20% of which are unsaturated fatty acids, including linoleic and oleic acids [3]. Carbohydrates include polysaccharides with functional properties (emulsifying, foaming, water/oil retention) and concurrent antioxidant activity [4]. It is worth emphasizing the presence of minerals, in particular P, Fe, and Ca, as well as volatile oils, with thymoquinone and thymol as the main biologically active substances [1]. The thymoquinone content in the essential oil can be as high as 30–48% [1]. Other active compounds in black cumin seeds include many alkaloids, saponins, flavonoids, terpenes, tocopherols, and phytosterols. Thymoquinone has been the subject of many studies indicating its antidiabetic, hypolipidemic, anti-inflammatory, neuroprotective, or anti-cancer activity [4,5]. The antidiabetic properties of thymoquinone have been extensively tested in an animal model [6]. Thymoquinone supplementation in humans may reduce oxidative stress and promote the proliferation of pancreatic beta cell integrity, thus leading to improved insulin secretion, reduction of intestinal glucose absorption, and inhibition of hepatic gluconeogenesis [7].

Studies showed that the inclusion of black cumin oil in the form of capsules in the diet was associated with a significant reduction in inflammatory markers in obese pre-diabetic subjects [8]. A meta-analysis performed by Mohit et al. [5] revealed that black seed contributed to a significant reduction in the values of inflammatory and oxidative stress markers, i.e., C-reactive protein (CRP) and malondialdehyde (MDA) in adults. Therefore, it seems that black seed can be used in the diet therapy of inflammatory diseases, such as type 2 diabetes or metabolic syndrome. Inflammation is associated, i.e., with the increased production of reactive oxygen species (ROS). ROS are by-products of oxygen consumption and cellular metabolism. According to current knowledge, increased amounts of ROS contribute to cancer development in an epigenetic and genetic way [9]. The oxidative stress-induced development of breast cancers, contributed by obesity and chronic inflammation, is well known [10,11].

Antioxidants also play an important role in the food industry. They influence the oxidative stability of fats present in various products, the oxidation of which leads to a deterioration of food quality and the formation of compounds harmful to human health [12]. The effect of the addition of black cumin extract/oil on oxidative stability was investigated both in a meat product, i.e., patties stuffed with beef [13], and in a product based on vegetable oil, i.e., mayonnaise [12]. In each of the cited experiments, the black cumin supplement improved the oxidative stability of the products. Black cumin is widely used in the food industry as a spice and a raw material for the production of oil. Black cumin protein concentrates with their foaming properties appear to be a potentially useful ingredient in food technology [14]. The cake, i.e., the residue from pressing various oils, is a waste product. Due to the nutritional properties of cake, which is a good source of fiber and antioxidants, ways are sought to reuse this by-product in human nutrition. So far, inter alia, coconut pomaces [15], or flaxseed cake [16] have been used in the production of functional pasta with improved nutritional composition. Black cumin seed press cake was previously used, i.e., for the development of non-dairy beverages fermented with Kefir grains [17].

Pasta is a popular frequently consumed and readily available food item. It can be an excellent food matrix to incorporate ingredients with functional properties. So far, attempts have been made to obtain semolina pasta enriched with a mixture of black cumin and a fiber preparation, but for fear of worsening sensory features, the use of black cumin has been abandoned [18] (there are no reports in the literature on the possibility of fortifying durum wheat pasta with black cumin cake, while gluten-free bread, with the addition of this type of cake, was successfully produced (0–10%)) [19]. There were also sponge cakes produced with the addition of black cumin seed powder [20].

The purpose of the study was to determine the possibility of using black cumin cake to obtain pasta with improved nutritional value and functional technological quality.

## 2. Materials and Methods

### 2.1. Characteristics of Raw Materials

Durum semolina (Julia Malom, Kunszállás, Hungary) and black cumin cake were used for the production of pasta. The cake was obtained by pressing oil from black cumin seeds (ViVio, Brzozów, Poland). The screw press with a capacity of 18–25 kg h^−1^, screw speed of 1500 rpm, and 10 mm dies were used for cold pressing (<50 °C) (DUO; Farmet, Česká Skalice, Czech Republic). The cake was ground and designated for further research. The control sample (CON) was made of durum semolina. The other samples were enriched with different doses of the cake: 5, 10, 15, 20, and 25% (samples BC5, BC10, BC15, BC20, and BC25, respectively). The detailed model of the experiment is presented in Table 1.

### 2.2. Pasta Preparation

A MAC-30S Lab pasta extruder (ItalPast, Fidenza, Italy) and an EAC30-LAB pasta dryer (ItalPast, Fidenza, Italy) were used for the production of pasta (ribbon-shaped) in semi-technical conditions. Before pressing, a 4 kg sample of raw materials was mixed for 15 min under pressure and then under a vacuum pressure of 0.085 MPa. The rotational speed of the pasta extruder screw was 48 rpm. The pasta samples were dried at a controlled temperature and humidity. Detailed conditions of this process were described by Sobota et al. [21].

### 2.3. Chemical Analysis

The analysis of the chemical composition was performed using the AACC and AOAC methods [22,23].

Moisture content was determined using the air-oven method (Method AACC 44-15A). The samples (3 g) were placed in a laboratory dryer and dried at 103 °C ± 1 °C to constant weight.

Ash content was determined using the AACC method 08–01. The samples were measured into ash dishes in amounts of 3 g and then placed in a muffle furnace at 550 °C. They were incinerated until light gray ash or constant weight was obtained (7 h). After cooling, the samples were weighed, and the ash contents were calculated.

Total protein content was determined using the KjeltecTM8400 apparatus (Foss Analytical AB, Höganäs, Sweden). The distillation was carried out in an automatic Kjeltec Auto kit by Tecator. The nitrogen content was converted to protein using the N × 5.7 conversion factor.

Total fat content was determined by continuous ether extraction in the Soxtec TM8000 apparatus (Foss Analytical AB, Höganäs, Sweden) using hexane as a solvent. The tests were performed in two replications.

Total dietary fiber (TDF) content, as well as insoluble dietary fiber (IDF) and soluble dietary fiber (SDF), were determined according to enzymatic methods (AACC 32–05, AACC 32–21, AOAC 991.43, and AOAC 985.29). 1 g dried samples were subjected to sequential enzymatic digestion by heat-stable α-amylase, protease, and amyloglucosidase. Megazyme enzymes and analytical procedures were used (Megazyme International Ireland Ltd., Wicklow, Ireland).

Digestible carbohydrate content was performed by calculation of the difference: 100 − (weight in grams (protein + fat + TDF + ash) in 100 g of dry matter of pasta or raw material).

### 2.4. Determination of the Content of Mineral Elements

The analysis of chemical elements was performed on a Varian model AA 240 FS equipped with a D2 lamp background correction system using an air-acetylene flame (air 13.5 L/min, acetylene 2.0 L/min, Varian, Ltd., Mulgrave, Australia). The measurement results were compared with the multielemental standard for GF AAS (CertiPUR^®^, Merck, Germany). A 1 g sample was digested with a mixture of HNO3: redistilled water (1:1). The samples were digested in a closed vessel high-pressure microwave digester (MARS X-press, USA) for 55 min. After cooling to room temperature, the suspension was filtered through Munktell filter paper (grade 390.84 g/m^2^, Germany) and diluted to 50 mL with distilled water. Then, the sample extracts were subsequently analyzed for Cd, Pb, Cu, Zn, Co, Cr, Ni, Mn, and Fe using a fast sequential atomic absorption spectrophotometer (Varian model AA 240 FS). The compounds were tested at the following wavelengths after the calibration process: Cu—324.8 nm, Zn—213.9 nm, Cr—357.9 nm, Mn—279.5 nm, and Fe—241.8 nm.

### 2.5. Total Content of Polyphenols and Flavonoids

Total polyphenol content was measured in accordance with Singleton and Rossi (Singleton and Rossi, 1965) using Folin–Ciocalteu reagent. A 0.1 mL sample was mixed with 0.1 mL of the Folin–Ciocalteu reagent and 1 mL of 20% (*w/v*) sodium carbonate and left in darkness for 30 min. The absorbance at 700 nm was measured using a Jenway spectrophotometer (6405 UV/Vis, England). Gallic acid (1–150 mg/L; R^2^ = 0.999) was used as a standard and the results were expressed in mg/g of gallic acid equivalent.

Total flavonoid content was determined using the modified method proposed by Quettier-Deleu et al. (Quettier-Deleu et al., 2000). The sample (2 mL) was mixed with 0.4 mL of a 5% (*w/v*) ethanolic solution of aluminum chloride. After 30 min in darkness, the absorbance at 405 nm was measured using a Jenway spectrophotometer (6405 UV/Vis, England). Quercetin (0.25–20 mg/L; R^2^ = 0.989) was used as a standard and the results were expressed in µg/g quercetin equivalents.

### 2.6. Antioxidant Activity

Radical scavenging activity of extracts (DPPH method) was measured using 2,2-diphenyl-1-picrylhydrazyl (DPPH) (Yen and Chen, 2002). The sample (0.5 mL) was mixed with 2 mL of a DPPH solution (0.025 g DPPH in 100 mL ethanol). The absorbance of the reaction mixture was determined using a Jenway spectrophotometer (6405 UV/Vis, England) at 515 nm. Trolox (6-hydroxy-2,5,7,8-tetramethylchroman-2-carboxylic acid) (10–500 mg/L; R^2^ = 0.983) was used as a standard and the results were expressed in mg/g Trolox equivalents.

### 2.7. Determination of the In Vitro Starch Hydrolysis Index (HI) and Glycemic Index (GI)

The procedure was previously described by Krawęcka et al. [24]. After in vitro digestion, the GOPOD method was used to determine the glucose content in the samples (mg glucose/g sample) (D-Glucose Assay Kit, Megazyme, USA), which was plotted as a function of time, and the areas under the hydrolysis curves (AUC) were calculated. Subsequently, the hydrolysis index (HI) was calculated as the ratio between the AUC of the sample and the AUC for the reference food (white bread). The glycemic index was predicted according to the equation:

GI (%) = 39.71 + 0.549(1)

### 2.8. Cooking Quality of Pasta Samples

Cooking time (CT) was measured according to Method AACC 16–50 [22]. 50 g of pasta was boiled in 500 mL of distilled water. Every 30 s, the pasta was removed and squeezed between two glass plates until the mealy core disappeared. The time needed for this process was assumed as the cooking time (CT).

Weight increase index (WII) was calculated by dividing the weight of the pasta sample after cooking by the weight of the uncooked pasta sample (50 g).

Cooking loss (CL, g/100 g d.m.) was determined by testing the dry matter content in water after cooking a 50 g pasta sample. The dry matter content in water was determined according to the AACC 44–15A method [22].

### 2.9. Texture Parameters

Before the determination of hardness (measurement of the maximum cutting force), the pasta samples were cooked for the optimal cooking time. The material (50 g) was heated in 600 mL of boiling distilled water. The pasta was cooked in a gentle boil using the previously determined cooking time (CT). Then, the pasta was drained and rinsed with a stream of distilled water (rinsing time—30 s; the amount of water—100 mL). The material prepared in this way was transferred to a vessel with distilled water at room temperature. Strength tests were carried out immediately after cooking and preparing the pasta. The determinations were made using the Zwick/Roell Z0.5 testing machine (maximum pressing force 500 N, ZwickRoell AG, Ulm, Germany) with the head travel speed of 10 mm·min^−1^. Cutting tests were performed using a Warner–Bratzler flat knife and testXpert II software. Measurements were performed in 15 replications.

### 2.10. Statistical Analysis

The obtained results were subjected to statistical analysis using the software program STATISTICA 13.1 (StatSoft ©, Inc. Tulsa, OK, USA). All experimental results were means (± S.D) from at least three assays. One-way analysis of variance (ANOVA) and Tukey’s post hoc test were used to determine the significance of differences between means. The results were statistically different for *p*-values ≤ 0.05.

## 3. Results and Discussion

### 3.1. Pasta Processing

The addition of black cumin cake influenced the pasta extrusion process. The increasing content of the by-product in the enriched pasta from 5 to 25% resulted in a decrease in the pressure values from 13.5 to 9.2 MPa in the BC5 and BC25 pasta samples, respectively. The pressure in the case of the CON sample was 13 MPa. Additionally, it was observed that the use of 20–25% of the functional component resulted in a slight reduction (approximately 3%) in the efficiency of the pasta press (Table 1).

### 3.2. Basic Chemical Analysis

The chemical composition of the pasta samples is shown in Table 2. The black cumin cake contained significantly (*p* ≤ 0.05) higher amounts of protein compared to the semolina, which explained the significant (*p* ≤ 0.05) increase in the content of this macronutrient in the black cumin cake-enriched pasta. The pasta with the largest 25% share of the cake contained 28% higher protein content than the control sample (100% durum semolina). Similar protein content in the raw material (27.70 ± 0.18%) was determined in wheat flour pasta enriched with 2.5, 5, and 10% of the by-product from chia oil extraction in a study conducted by Aranibar et al. [25]. An increase in protein content in durum semolina pasta enriched with coconut pomace was also observed by Sykut-Domańska et al. [15]. In the present study, the enriched pasta was characterized by significantly (*p* < 0.05) higher fat content compared to the control sample, which resulted from the relatively high fat content in the cake. Along with the increase in the dose of the black cumin cake, the ash content increased significantly (*p* ≤ 0.05). Similar results were achieved by Sethi, Nanda, and Bala [26] by incorporation of black rice bran into wheat pasta and by Aranibar et al. [25], who used chia cake to enrich pasta. Black cumin cake is a high-fiber material. The enriched pasta tested in the present study was characterized by significantly (*p* ≤ 0.05) higher values of total fiber, mainly the insoluble fraction. The pasta with the highest 25% addition of black cumin cake contained almost 3-fold higher amounts of TDF than the control pasta. The obtained results are consistent with the results reported by other authors, who observed an increase in the content of dietary fiber or crude fiber after the addition of cake for the production of functional cereal products. The trend was reported in the case of enrichment with fruit pomace (pineapple pomaces) [27] and oilseed raw material (flaxseed cake) [16]. The content of the soluble fraction (SDF) and the insoluble fiber fraction (IDF) were found to increase after supplementation with fruit pomace and oilseed cake, respectively. In the present study, the enhancement of the dietary fiber content in black cumin cake-enriched samples was especially evident in the case of the insoluble fraction. The estimated digestible carbohydrate content in the enriched pasta decreased, compared to the content in the control sample. A reduction in the content of digestible carbohydrates after introducing raw materials with a high content of insoluble dietary fiber into pasta was already observed in our previous studies [24]. Products with lower content of digestible carbohydrates, compared to the control, should induce a lower glycemic load (GL) and lower glycemic response, and in this way may have a positive effect on carbohydrate metabolism and diabetes prevention [24].

### 3.3. Determination of the Content of Mineral Elements

Vegetable by-products may contain anti-nutritional ingredients such as trypsin inhibitors, phytates, and tannins. These substances may adversely affect the bioavailability of minerals and protein digestion, and their content may decrease during technological processes, for example, oil extraction [28]. The observed increased ash content (Table 2) in the enriched pasta is synonymous with higher content of minerals. Black cumin cake supplementation had a particularly positive effect on the content of zinc, iron, and manganese in the products (Table 3). The content of zinc and manganese in the 25% enrichment variant increased almost twice, compared to the control sample, while the content of iron increased over 2.5 times. In the case of pasta products, a significant increase in mineral content after the addition of pomaces was observed, among others, in semolina pasta enriched with millet flour and carrot pomace [29]. The authors observed an increase in the content of calcium, iron, and zinc. Mineral homeostasis is of key biochemical importance to the metabolic activity and regulatory mechanisms in the body. Studies show that low zinc consumption increases the risk of type 2 diabetes [30]. The risk can be reduced by up to 20% by increasing zinc intake to over 13 mg/day [31]. Chromium, on the other hand, is involved in the metabolism of insulin, as shown in studies on patients with type 2 diabetes with a deficiency of this micronutrient [32].

### 3.4. Total Polyphenol and Flavonoid Content and Antioxidant Activity

Phenolic compounds, including a subgroup of flavonoids, are the most important components influencing the antioxidant activity [1]. Antioxidant protection is crucial, both in terms of maintaining the desired color and taste of food and in balancing the rate of production and release of free radicals in the human body. It is assumed that the high temperature extrusion (HTST) process may have a negative impact on heat labile compounds and cause the polymerization of some phenolic compounds as a result of high temperature and high pressure. Pasta is produced with the technology of low-temperature extrusion, which limits the inactivation of biologically active compounds [33].

The addition of black cumin cake caused a significant (*p* ≤ 0.05) increase in the total content of polyphenols and flavonoids (Table 4) in the uncooked products. In the raw pasta, a significant (*p* ≤ 0.05) increase in the total content of polyphenols and flavonoids was observed, even at the smallest 5% addition of black cumin cake. The content of flavonoids in the pasta with the highest content of black cumin cake (25%) was over 5 times higher than in the control pasta. The hydrothermal treatment process of pasta samples caused losses of polyphenols. After cooking, the content of polyphenols in the BC25 pasta decreased by as much as 62.5%. At the same time, it was found that the content of phenolic compounds in cooked products with the greatest addition of the cake (BC15, BC20, and BC25) did not differ significantly. Similar results were observed by Turco et al. (2019) in high-fiber pasta obtained in 100% from legume seed flour, where the hydrothermal treatment reduced the total content of polyphenols by 50–60% [34]. On the other hand, the hydrothermal treatment had a significant (*p* ≤ 0.05) positive influence on the content of polyphenols in the control sample. The pasta was cooked for a predetermined optimal cooking time. The BC5 and BC10 samples were characterized by the longest cooking time (7 min and 6’30 min, respectively) (Table 5). As a result of the hydrothermal treatment, there was a noted significant (*p* ≤ 0.05) increase of flavonoids. It should be emphasized that plant polyphenolic compounds can be present in food in free or bound and soluble or insoluble forms. Loss of polyphenols in cooked pasta may be due to their solubility and leaching into solution during cooking. As reported by Carcea et al. [35], thermal treatment causes losses of water-soluble polyphenols, at the same time increasing the content of bound polyphenols, which explains our results. Increase in the polyphenol content as a result of the thermal treatment of cereal products may result from the partial hydrolysis of indigestible polysaccharides. Phenolic compounds built into fiber structures are called co-passengers. In their native form, they have limited bioavailability and concurrently show lower antioxidant potential. The disintegration of the fiber matrix leads to an increase in the content and antioxidant potential of polyphenols. Research has shown that heat treatment alone increases the polyphenol content in wheat and oat bran [36]. Polyphenols can also interact with pasta ingredients (the gluten present in the flour and egg whites, if used). Their stability is also influenced by the method of drying the pasta [37]. This explains the obtained results and the possibility of a different influence of the pasta cooking process on the polyphenol content in the CON sample and the BC25 sample. Additionally, literature data indicate that thermal treatment may have both positive and negative effects on the polyphenols contained in pomaces [38]. In a study conducted by Vodnar et al. [38], extracts from apple and white grape pomace were characterized by higher total polyphenol content after heat treatment. Researchers are constantly looking for the best methods of processing raw materials before enriching pasta products to ensure the smallest possible loss of active substances during the processes used [37].

There is an inverse relationship between the occurrence of diseases and the delivery of antioxidants inhibiting lipid peroxidation, chelating metal ions, and scavenging free radicals in the organism [1]. The DPPH method used to determine the antioxidant activity of products is based on hydrogen donation ability. In this study, it was found (Table 4) that the addition of black cumin cake had a significant (*p* ≤ 0.05) effect on the antioxidant activity of raw pasta. The antioxidant activity in the uncooked pasta with the 25% addition of BC was almost twice as high as in the control sample. In the case of the cooked samples enriched at the levels of 15, 20, and 25%, there was a slight, albeit significant, difference (*p* ≤ 0.05) compared to the control semolina pasta. These samples contained the highest amounts of polyphenols, which was reflected by an increase in antioxidant activity. Increased antioxidant activity (both DPPH and FRAP assays) after incorporating black rice bran into wheat pasta was linked by Sethi et al. [26] to the presence of dark-colored anthocyanins, also present in black cumin seeds. Olive pomace-enriched semolina pasta obtained by Simonato et al. [39] was characterized by higher antioxidant activity (DPPH and ABTS assays) in both raw and cooked samples, and the cooking process of the pasta had a negative effect on the antioxidant activity. A statistically insignificant decrease in the antioxidant activity after cooking the pasta was noted in the present study.

### 3.5. In Vitro Starch Hydrolysis Index (HI) and Glycemic Index (GI)

Foods containing carbohydrates can be classified according to their potential effect on postprandial blood glucose levels, which can be assessed as a low, medium, and high glycemic index (GI) [40]. The in vitro starch hydrolysis index (HI) and glycemic index values (GI) of the pasta are shown in Table 6. All the pasta samples had a low glycemic index value; moreover, the fortification of the pasta with black cumin resulted in a lower glycemic index. The predicted GI indicated the same trend as starch hydrolysis, and the lowest HI (11.46% and 10.8%) and GI values (46.0 and 45.64) were recorded for samples BC20 and BC25 (*p* ≤ 0.05), respectively. The highest level of the *Nigella sativa* cake supplementation resulted in an approximately 4% decrease in GI relative to the control. Pasta has been proven to be a carbohydrate product with a more favorable effect on glycemia than bread, which was confirmed by our study, as even the control sample had a low glycemic index (47.57) [41]. The digestibility of starch contained in foods, especially those treated with different types of heat, depends on the other components present in the food, including proteins, fats, fiber, or polyphenols. Therefore, attempts are being made to lower the glycemic index of products by adding different ingredients to the basic recipe [42,43]. In turn, there are at least several possible reasons why black cumin fortification lowered the glycemic index of the pasta. Black cumin is rich in protein, fats, and fiber; therefore, using this raw material for pasta supplementation is reasonable because each of these ingredients can affect GI [44]. Starch can interact with lipids and proteins through electrostatic and hydrophobic interactions, which increase the ordering of the starch structure and slow down its digestibility. These interactions play a key role in modulation of digestibility and can occur in various conditions [45]. The availability of starch to digestive enzymes may also be reduced by the presence of fiber and the formation of a viscous protein–fiber–starch network that can trap starch granules, limiting the release of glucose [46]. Black cumin is also rich in antioxidants that may have functional effects on digestion and carbohydrate absorption [44]. Polyphenol compounds, whose contents increased with the increase in the share of the black cumin cake, inhibit amylolytic enzymes and delay glucose absorption, contributing to lower postprandial glycemia [47]. Given the great interest in low glycemic index products expressed by subjects who often struggle with various conditions, the results obtained are important and provide a basis for further research in this field to confirm the properties of black cumin.

### 3.6. Cooking Quality

Previous studies indicate that the addition of raw materials with high dietary fiber content may extend the cooking time [48]. However, according to the literature, some high-fiber preparations and raw materials such as inulin, durum bran, and bean flour caused the opposite tendency [49]. Although the 5% enrichment of durum semolina with the black seed pomace caused a significant (*p* ≤ 0.05) extension of CT in our study, a successive shortening of CT was observed, along with the further increase in the cake addition dose (Table 6). An increase in the proportion of pomace, which is particularly rich in insoluble dietary fiber, may contribute to the disintegration of the gluten network and thus facilitate the swelling and gelatinization of starch. The cooking time for the BC25 sample was the same as for the CON pasta. Along with the increase in the black cumin cake dose, the cooking loss values of the cooked pasta samples increased significantly (*p* ≤ 0.05). Similar dependencies were noted by Simonato et al. [39] during the production of semolina pasta enriched with olive pomace. The incorporation of a high-fat and high-fiber additive into the pasta resulted in reduced CT and increased cooking loss, swelling index, and water absorption. As suggested by Matsuo et al. [50], fatty acids produced in the pasta storage process inhibit the swelling of starch granules, which may cause CT extension. In our research, the loss of dry matter in the pasta enriched at the level of 10–25% exceeded 8%. According to Teterycz et al. [51], in good quality pasta, this parameter should not exceed 8%, but Wu et al. [52] gives a value of up to 10%. The dry matter loss depends largely on the shape and size of the pasta. Smaller forms with high specific surface area may be prone to more intensive leaching of dry matter components (mainly starch) from the product. One of the most important elements that affects the quality characteristics of pasta is the stable combination of gluten proteins that make up the product matrix. The increase in dry matter loss could be caused primarily by the higher fiber content and the dilution of the gluten protein network. The effect of IDF on the interaction with gluten depends on the degree of hydration and may induce changes in the structure of gluten [53]. IDF disintegrates the protein matrix. A high degree of comminution of high-fiber ingredients is conducive to the maintenance of the continuity of the gluten network and may reduce the amount of dry matter losses. Increasing the degree of bagasse crushing may simultaneously expose co-passengers (for example, polyphenols) embedded in the fiber structures and their more intensive transfer to the water when cooking the product [35]. Moreover, as a result of the thermal treatment, some of the complexed phenolic compounds can be released and get into the solution [54]. The analysis of the total content of polyphenols and flavonoids (Table 4) revealed that the hydrothermal treatment resulted in a partial loss of these compounds. In a chromatographic analysis, Balli et al. [37] found that the thermal treatment of pasta enriched with grape marc (7%) and olive pomace (7%) resulted in the transfer of anthocyanins, i.e., compounds responsible for the color, into the solution. With the increase in the share of high-fiber raw material, the weight gain of pasta during cooking decreased significantly (*p* ≤ 0.05) (samples BC5, BC10, BC25). This is likely related to the competition for water absorption between fiber and starch. Similar observations were made by Padalino et al. [55], examining durum wheat pasta enriched with tomato pomaces.

### 3.7. Texture Parameters

The cooking time has the greatest influence on the texture of cooked pasta [56]. Each pasta sample was cooked at an individually assigned cooking time to exclude the variable effect of the hydrothermal treatment, as suggested by Duda et al. [57]. The samples enriched with black cumin cake at the level of 5–10% showed significantly (*p* ≤ 0.05) lower hardness (F*_max_*) (Table 6) compared to the control. In turn, the samples with an increased share of the functional additive (at the level of 15, 20, and 25%) were characterized by hardness values similar (*p* ≤ 0.05) to that of the control pasta (4.56 N). The functional additive used in the present study was not only high in protein but also high in fiber. As reported by Espinosa-Solis et al. [58], semolina durum pasta enriched with unripe apple flour had increased hardness, greater than in the control sample (100% semolina) and pasta with the addition of oat bran. Hardness/firmness is mainly affected by the protein fraction. In the present study, the increase in the dose of black cumin cake and, at the same time, the reduced share of semolina were accompanied by a decrease in the content of gluten proteins responsible for the formation of a stable protein matrix. Moreover, the significant (*p* ≤ 0.05) increase in the IDF content and the affinity of the fiber to water could have made the product harder. In the literature, the effect of an additive containing high content of proteins other than gluten proteins and/or fiber on the increase in the hardness or firmness (expressed indirectly by hardness) parameter was observed in the case of pasta enriched with cricket powder [57] and pasta enriched with hemp seed cake [51]. The analysis of another parameter, i.e., cutting work required to cut the sample completely, showed that the highest values were obtained for the control sample and the sample enriched with 25% of black cumin cake (*p* ≤ 0.05). As explained by Dziki et al. [56], the higher values of both parameters for the control sample (100% durum semolina) may result from the high content of gluten in the durum wheat, which in turn is associated with the lower losses of dry matter during cooking and thus the greater firmness of the product.

## 4. Conclusions

With its documented properties, black cumin is a raw material worth paying attention to in the context of functional food design. Black cumin cake enhanced the protein, fat, ash, and dietary fiber in pasta. It also improved the antioxidant properties of the products and significantly influenced the content of valuable minerals. As the cake dose increased, a significant reduction in the starch hydrolysis index (HI) and glycemic index values (GI) of the pasta was noted. Due to the large loss of dry matter during cooking, the main technological challenge in designing functional pasta enriched with black cumin cake is to strengthen the structure of the pasta, which will ensure a better culinary quality of the product and reduce the loss of bioactive substances. The recommended addition of black cumin cake to the pasta should not exceed 20%.

## Figures and Tables

**Table 1:** Model of the experiment.

Samples	Raw Materials (%)		Pressure (MPa)	Barrel Temp. (°C)	Extruder Output (kg h^−1^)
	Semolina Durum	Black Cumin Cake			
**CON**	100	0	13	27.7	32.22
BC5	95	5	13.5	27.5	30.24
BC10	90	10	11	27	30.96
BC15	85	15	9.5	27.1	30.96
BC20	80	20	9.5	26.9	29.88
BC25	75	25	9.2	26.9	28.44

Explanation: CON—control sample; BC—pasta with black cumin cake.

**Table 2:** Basic chemical composition of raw material and pasta samples.

Samples	Moisture	Protein	Fat	Ash	TDF	IDF	SDF	Digestible Carbohydrate
	(%)				% d.m.			
Raw materials *								
Semolina durum	9.38 ^b^ ± 0.07	13.41 ^a^ ± 0.02	0.58 ^a^ ± 0.01	0.94 ^a^ ± 0.01	4.25 ^a^ ± 0.35	2.22 ^a^ ± 0.3	2.03 ^a^ ± 0.05	80.82
Black cumin cake	5.88 ^a^ ± 0.14	24.53 ^b^ ± 1.73	18.38 ^b^ ± 0.15	5.19 ^b^ ± 0.14	51.90 ^b^ ± 4.9	48.16 ^b^ ± 7.42	3.53 ^a^ ± 2.52	0.2
**Pasta samples**								
CON	5.39 ^a^ ± 0.01	12.76 ^a^ ± 0.1	0.13 ^a^ ± 0.01	1.00 ^a^ ± 0.02	5.39 ^a^ ± 0.27	2.37 ^a^ ± 1.15	3.02 ^a^ ± 0.12	80.72
BC5	5.01 ^ab^ ± 0.18	13.46 ^b^ ± 0.17	0.23 ^a^ ± 0.01	1.23 ^b^ ± 0.02	7.47 ^b^ ± 0.39	4.14 ^ab^ ± 0.35	3.34 ^ab^ ± 0.04	77.61
BC10	5.33 ^a^ ± 0.01	14.25 ^c^ ± 0.04	0.55 ^b^ ± 0.11	1.47 ^c^ ± 0.02	9.03 ^c^ ± 0.39	5.32 ^b^ ± 0.04	3.66 ^ab^ ± 0.43	74.70
BC15	5.24 ^ab^ ± 0.09	15.07 ^d^ ± 0.06	1.66 ^c^ ± 0.14	1.73 ^d^ ± 0.01	11.12 ^d^ ± 0.11	7.15 ^c^ ± 0.44	3.97 ^ab^ ± 0.34	70.42
BC20	5.31 ^a^ ± 0.05	15.96 ^e^ ± 0.01	3.65 ^e^ ± 0.02	2.03 ^e^ ± 0.01	12.92 ^e^ ± 0.21	8.61 ^c^ ± 0.01	4.31 ^b^ ± 0.19	65.44
BC25	4.94 ^b^ ± 0.19	16.41 ^f^ ± 0.01	3.31 ^d^ ± 0.04	2.20 ^f^ ± 0.02	14.79 ^f^ ± 0.5	10.43 ^d^ ± 0.93	4.36 ^b^ ± 0.43	63.29

Explanation: IDF—insoluble dietary fiber; SDF—soluble dietary fiber; TDF—total dietary fiber; CON—control sample; BC—pasta with black cumin cake. Data are presented as mean (*n* = 3) ± standard deviation. Data value of each parameter with different superscript letter (a–f) in the columns are significantly different (Tukey test, *p* ≤ 0.05). *—Statistically significant differences in the raw materials were determined excluding the series of results for the pasta samples.

**Table 3:** Chemical elements in the raw materials and pasta samples.

Raw Material	Concentration				
	Cu	Zn	Mn	Fe	Cr
	mg/kg of d.m.				
Semolina Durum	3.09	12.03	8.06	24.61	nd
Black Cumin Cake	15.41	44.62	33.47	210.48	0.96
**Pasta Samples**					
CON	4.12	12.37	7.72	22.83	0.11
BC5	6.01	15.18	9.59	31.84	0.11
BC10	6.13	16.37	10.88	39.93	0.21
BC15	6.75	19.30	12.13	51.04	0.21
BC20	6.97	20.80	13.62	55.00	0.21
BC25	8.51	22.06	14.50	58.62	0.32

Explanation: CON—control sample; BC—pasta with black cumin cake; nd—not detected; d.m.—dry matter.

**Table 4:** Total polyphenol and flavonoid content and antioxidant activity of pasta.

Sample	Polyphenols mg GAE/g		Flavonoids µg QE/g		Radical Scavenging Activity (DPPH) mg TEAC/g	
Raw materials *						
**Semolina durum**	0.32 ^a^* ± 0		2.20 ^a^* ± 0		0.98 ^a^* ± 0.05	
Black cumin cake	2.93 ^b^* ± 0.05		272.57 ^b^* ± 2.47		3.08 ^b^* ± 0.06	
**Pasta samples**	**Uncooked**	**Cooked**	**Uncooked**	**Cooked**	**Uncooked**	**Cooked**
CON	0.02 ^aA^ ± 0.02	0.28 ^aB^ ± 0	15.05 ^ab^ ± 6.49	n.d.	1.04 ^aA^ ± 0.06	0.96 ^aA^ ± 0.01
BC5	0.16 ^bA^ ± 0.06	0.37 ^aA^ ± 0.03	16.70 ^bc^ ± 1.30	n.d.	1.42 ^bA^ ± 0.10	0.96 ^aA^ ± 0.01
BC10	0.38 ^cA^ ± 0.01	0.43 ^abA^ ± 0.04	29.77 ^c^ ± 1.62	n.d.	1.60 ^bcA^ ± 0.05	1.02 ^aA^ ± 0.03
BC15	0.64 ^dA^ ± 0	0.58 ^cA^ ± 0.03	59.17 ^dB^ ± 1.04	1.06 ^aA^ ± 0.58	1.76 ^cdA^ ± 0.02	1.23 ^bA^ ± 0.01
BC20	0.89 ^eA^ ± 0.01	0.62 ^cA^ ± 0.07	67.39 ^deA^ ± 4.48	14.50 ^bA^ ± 3.89	1.80 ^cdA^ ± 0.10	1.33 ^bA^ ± 0.06
BC25	1.12 ^fA^ ± 0.02	0.70 ^cB^ ± 0.06	79.72 ^eA^ ± 4.54	34.17 ^cA^ ± 0.45	1.97 ^dA^ ± 0.03	1.35 ^bA^ ± 0.07

Explanation: CON—control sample; BC—pasta with black cumin cake. Data are presented as mean (*n* = 2) ± standard deviation. Values of each parameter with different uppercase superscript letters (A–B) in the rows are significantly different (Tukey’s test, *p* ≤ 0.05). Values of each parameter with different lowercase superscript letters (a–f) in the columns are significantly different (Tukey’s test, *p* ≤ 0.05). *—Statistically significant differences in the raw materials were determined excluding the series of results for the pasta samples.

**Table 5:** Cooking quality and texture of pasta samples.

Pasta Samples	Cooking Time (min)	Cooking Loss (% d.m.)	Cooking Weight Increase	F*_max_* (N)	Cutting Work (mJ)
CON	5 ^a^	6.00 ^a^ ± 0.95	2.30 ^ab^ ± 0.04	4.56 ^b^ ± 0.34	0.913 ^d^ ± 0.06
BC5	7 ^c^	6.67 ^ab^ ± 0.11	2.39 ^bc^ ± 0.03	3.89 ^a^ ± 0.25	0.778 ^a^ ± 0.04
BC10	6′30 ^bc^	8.26 ^bc^ ± 0.17	2.52 ^c^ ± 0.01	4.00 ^a^ ± 0.13	0.799 ^ab^ ± 0.02
BC15	6 ^bc^	8.25 ^bc^ ± 0.47	2.34 ^abc^ ± 0.12	4.08 ^ab^ ± 1.10	0.816 ^b^ ± 0.02
BC20	5′30 ^ab^	8.61 ^c^ ± 0.08	2.27 ^ab^ ± 0.01	4.27 ^ab^ ± 0.15	0.855 ^c^ ± 0.03
BC25	5 ^ab^	9.04 ^c^ ± 0.16	2.17 ^a^ ± 0.03	4.39 ^ab^ ± 0.17	0.878 ^cd^ ± 0.03

Explanation: % d.m—% of dry matter; CON—control sample; BC—pasta with black cumin cake. Data are presented as mean (*n* = 2) ± standard deviation for CT, CL, and WII measurements and (*n* = 3) for F*_max_* and cutting work. Data value of each parameter with different superscript letter (a–d) in the columns are significantly different (Tukey test, *p* ≤ 0.05).

**Table 6:** Hydrolysis index of starch (HI) and glycemic index (GI) of the pasta samples.

Pasta Samples	HI (%)	GI
CON	14.32 ± 0.89 ^a^	47.57 ± 0.49 ^a^
BC5	12.88 ± 0.52 ^ab^	46.78 ± 0.29 ^ab^
BC10	12.65 ± 0.3 ^ab^	46.65 ± 0.16 ^ab^
BC15	12.44 ± 0.26 ^ab^	46.54 ± 0.14 ^ab^
BC20	11.46 ± 0.1 ^c^	46.00 ± 0.06 ^c^
BC25	10.8 ± 0.71 ^c^	45.64 ± 0.39 ^c^

Explanation: HI—hydrolysis index of starch; GI—glycemic index values; CON—control sample; BC—black cumin cake. Data are presented as mean (*n* = 2) ± standard deviation. Data values of each parameter with different superscript letters (a–c) are significantly different (Tukey’s test, *p* ≤ 0.05).

## Data Availability

Not applicable.

## Sample Availability

Samples of the pasta are available from the authors.
